# Brazil must complete the cycle in vaccine development

**DOI:** 10.1590/0102-311XEN043024

**Published:** 2024-06-21

**Authors:** Breno Castello Branco Beirão

**Affiliations:** 1 Universidade Federal do Paraná, Curitiba, Brasil.

Editors of CSP,

In mid-2019, before the COVID-19 pandemic, an article was published in CSP which discusses public health issues that are important today: *Vaccines for Neglected and Emerging Diseases in Brazil by 2030: the “Valley of Death” and Opportunities for RD&I in Vaccinology 4.0* ^1^. Despite the time lag between the publication and this commentary, the referred article is as necessary today as it was at the time of publication. The authors state with up-to-date correctness the steps forward for local research in vaccinology if it is to be useful for our country.

As the article mentions, the development of immunobiologicals from preclinical to clinical stages has a “valley of death”. In Brazil, there have been recent advances in that front, following investments made via research grants and local governments in local laboratory infrastructure to support clinical development of vaccines ^2^ ^,^ ^3^. Nevertheless, this should be only the first step. Much work must be done nationally if Brazil is to be prepared for current health challenges.

Several diseases affecting Brazilians could be preventable by vaccination. The recent success with dengue vaccines ^4^ should stimulate vaccine research against other arboviruses (Zika and chikungunya), against leishmaniasis, Chagas’s disease, *Sporothrix brasiliensis*, and many other pathogens that afflict the Brazilian population. It is necessary to progress within the country since research on these pathogens depends on the nations that suffer from them ^5^.

In the article, the authors depicts how to progress in vaccine development locally, of which I especially endorse the need for driving vaccinology research all the way through to clinical stages, instead of falling in the “valley of death” after preclinical work. Cost/benefit analyses are clearly in favor of vaccine research for the benefit of public health, but this depends mostly on it actually impacting the population disease burden ^6^ ^,^ ^7^. To this end, vaccine research must reach clinical trials ^8^ ^,^ ^9^. This is costly, but as mentioned, it is cost-effective. Brazil already has a strong background in preclinical immunobiological research and is ripe to collect the results in the form of vaccines and biological treatments being translated into the Brazilian Unified National Health System (SUS).

According to the authors of the referred article, funding is one of the measures to drive Brazilian science into a mature phase of clinical research. Financial support is always necessary, but maybe hard to implement now. The percentage of gross domestic product invested in science and research in Brazil is reasonable for a middle-income country. Research support in Brazil should be more constant and less politically dependable ^10^ ^,^ ^11^, but I advocate that research institutions must make more of an effort of delivering outputs that are directly beneficial to the public to be able to demand more of the gross domestic product (GDP). Personally, I believe us - Brazilian researchers - must always bear in mind that poverty is still the widespread reality in our country ^12^, and as such it is up to this date the most relevant issue of the Nation. Measures that positively impact poverty - both direct and immediate measures - must remain as the priority of the day until that problem is resolved - starving people cannot wait for science.

Investment is a basic need for implementing novel health products, certainly, but even simpler actions have great impact. A word of praise should be given to the effort of the Brazilian Health Regulatory Agency (ANVISA) of receiving vaccine dossiers in a “continuous flow”, thus easing the bureaucratic effort faced by national research institutions that have no or little expertise (and dedicated personnel) in regulatory issues ^13^. However, more could be done. Private investment should be a constant in research, and Brazil lags in this issue ^14^. Undoubtedly, the private pharmaceutical sector in the country must move towards more innovation ^15^, but this will only come with a simpler and more stable regulatory regime and strong de-bureaucratization of the interaction of companies with universities and public institutions ^16^. This would also open the path for startups and smaller companies which currently cannot face the barrier to entry in such a high-investment industry as is the health sector ^8^.

Indeed, de-bureaucratization is in the agenda not only for health R&D (research and development), but for Brazil as a whole ^17^ ^,^ ^18^. Public organizations must understand their role in national development, and this is not currently possible ^16^. Anticorruption measures bear a heavy load on public research while doing little to neutralize ongoing high-level corruption. On the other hand, public organizations often do not fulfil their societal roles and time is lost in waiting for top-down resolutions ^19^. Public change will happen in public deeds.

In summary, I believe the struggle of the pandemic has had the positive impact of pushing Brazilian R&D towards the goals of the cited article, but now is the time to keep up the momentum to be able to come close of reaching the goals set in the article.
